# Interdisciplinary Approach to Improve Agri-Food Safety and Quality

**DOI:** 10.3390/ijerph19148801

**Published:** 2022-07-20

**Authors:** Francesca Serio

**Affiliations:** Department of Biological and Environmental Sciences and Technology, University of Salento, 73100 Lecce, Italy; francesca.serio@unisalento.it

The agri-food sector is very broad and includes a whole series of steps in the supply chain ranging from agricultural production to the processing and marketing of final products.

Food is the central axis of human relations and carries different meanings that arise from the history and cultural heritage of the inhabitants of a particular area. The food tradition thus becomes part of the particularity of a territory. Cultural richness can be associated, for example, with the traditional use of wild plants that are important food for rural populations. In the past, peasant families used these resources to satisfy their food needs as amply demonstrated by historical texts; nowadays, man no longer needs to collect herbs to survive, but experiences pleasure of getting something “natural” that allows him to get closer to nature, to rediscover ancient and more intense flavors than those deriving from cultivation techniques deriving from food globalization. Rediscovering old rural habits can be associated with sustainable agriculture models and the safeguarding of territories [1].

At the same time, concerns about the health of consumers and the safety of farmers, as well as the strong social pressures for the use of environmentally friendly production practices, have urged scientific research towards the study of the nutraceutical and health properties of many active substances in food.

The meaning of “quality” in the context of food hygiene has also undergone an important evolution; we no longer require only the absence of contaminants, but also the presence of elements with beneficial effects for our health. Therefore, the more modern concept of health promotion has been added to the concept of prevention.

This new need has led to the development of increasingly sensitive analytical approaches aimed at the molecular characterization of agri-food products along the entire supply chain; in this context, metabolomics quickly emerged as an effective tool for use in the food and nutrition sciences. Among the most used techniques, NMR spectroscopy is able to provide the highest performance in terms of detectability of different metabolites.

Among the most widely used analytical techniques, NMR spectroscopy is a high-performance, non-invasive, highly reproducible and quantitative technique that provides detailed information on the molecular basis of complex mixtures. This technique has shown great success in the evaluation of health and safety aspects of food and has been able to provide further information on the state of genuineness and quality of food.

In the study by Girelli et al. [2], an NMR-based metabolomic approach was applied to four local varieties of chicory; this approach made it possible to improve the knowledge on their nutraceutical value by identifying the bioactive compounds that can bring about possible benefits for human health. This interdisciplinary article, which characterized local varieties of *C. intybus*, made it possible to determine their potential usefulness, as well as to define specific qualitative-nutritional characteristics. The results obtained from this and other works that exploit holistic approaches could improve knowledge on the products of the agri-food chain, also highlighting their nutraceutical properties. Food quality and safety awareness has gained significant importance among consumers, who are driven to consume foods and beverages that provide healthy nutraceutical nutrients, bioactive compounds. Since the use of an interdisciplinary approach is essential, this Special Issue also hosts articles that demonstrate the important role that the integrated and multidisciplinary perspective can play in relation to food safety issues with a broader view of what is health promotion.

Grasso et al. [3] carried out a chemical characterization and quantification of AgNPs and dissolved Ag, in processed canned seafood products, a product that makes up a significant contribution to the diet (unsaturated fatty acids, protein and different micronutrients), with the emerging technique of Single Particle-Inductively Coupled Plasma (spICP-MS). This technique allows the determination of particle number-based concentration, with the rapid simultaneous characterization of its elemental composition, number of particles, size and size distribution, in addition to the dissolved concentration. Some aspects related to diet are becoming increasingly important: consumers are moving towards a holistic approach to nutrition; they are thinking about prevention, aging healthily and sustainability; and eating habits are becoming increasingly personalized. We choose foods that can bring health benefits, as they contain substances that have positive effects on the balance and wellbeing of the body.

In this context, the presence of various bioactive components such as polyphenols (e.g., chlorogenic acid, cynarin, luteolin 7-O-rutinoside, luteolin 7-O-glucoside and 1,5-di-O-caffeylquinic acid), inulin, vitamin C, vitamin K and some minerals such as calcium, iron and zinc, in the artichoke plant, has generated considerable interest.

According to some researchers, polyphenols extracted from artichoke leaves could contribute to the prevention and management of some types of cancer. Other in vitro and in vivo studies demonstrated the ability of some bioactive compounds present in the artichoke to inhibit the angiogenesis and/or proliferation of some tumor cell lines, to influence the cell viability of the human leukemic K562 cell line and to induce the apoptotic pathway in human colorectal cancer DLD1 cells.

Villarini et al. [4] evaluated the cytotoxic, genotoxic and apoptotic activities, as well as the cell growth effect, of ALEs on human colon cancer HT-29 and RKO cells. ALEs have been characterized by their content in chlorogenic acid, cynaropicrin and caffeylquinic acids. HT-29 and RKO cells were used for in vitro tests (i.e., evaluation of cytotoxicity and genotoxicity, cell cycle analysis, induction of apoptosis). ALEs rich in cinaropicrin, caffeylquinic acids and chlorogenic acid were shown to be able to influence HT-29 and RKO colon cancer cells by inducing favorable biological effects, highlighting the potential cancer chemotherapy activities of ALEs including cell cycle disruption, the activation of the mitochondrial pathway of apoptosis and the induction of genotoxic effects probably mediated by the induction of apoptosis. Therefore, food safety and quality management systems are the most important goals to achieve in order to protect the health and wellbeing of people.

The approach to quality management in agri-food chains has also undergone important evolutions over time, passing from the conformity of the finished product according to quality standards downstream from the supply chain, to a broader concept that provides for quality control relating to the entire production process. The introduction of the HACCP model (Hazard Analysis and Critical Control Points) in the 1990s already went in this direction. More recently, the integrated control of agri-food chains has also included that relating to drinking water.

With regard to drinking water, current legislation requires compliance with the minimum requirements of sanitary quality and physical, chemical, microbiological and radiological quality at the point where the water is available for consumption. Even the most recent EU directive (2020/2184), currently being transposed by the Member States, follows this same direction.

The quality of drinking water is generally guaranteed by the good quality of water resources, as well as by a set of control measures and advanced technologies. The promotion of a new holistic approach that shifts the focus from the retrospective control of distributed water to the prevention and management of risks in the drinking water supply chain, as found in the model of the Water Safety Plans (WSP) developed by the World Organization of Health, has become fundamental. The WSPs are based on the assessment and management of the risk associated with each phase that makes up the water supply chain to ensure the protection of water resources and the reduction of potential health risks in drinking water.

If applied to small drinking water plants (water kiosks, third-range vegetable processing companies), it is possible to move from a management mainly focused on verifying the conformity of the final product to the creation of a risk assessment and management system that covers the entire water supply chain. In these cases, the model provides for a greater commitment in the analysis of the incoming water, the local intended use and the possibilities of managing the containment of the dangers to which it is exposed, but it is an approach that has demonstrated a concrete effectiveness in identifying and mitigating the dangers of altering the water quality [5].

Food quality and safety management systems are therefore the most important objectives to be achieved in order to protect people’s health and wellbeing. Therefore, it is necessary to adopt the most sensitive analytical methods in order to detect the composition and the relative chemical–physical properties of food matrices.

Food safety and food security are two different concepts that intersect each other and deeply influence the quality of human life. On the one hand, food safety is a complex of aspects concerning the production, handling, preparation and storage of food capable of ensuring the healthiness of products and avoiding the onset of diseases in humans. On the other hand, food security, as defined by the FAO, allows for physical, economic and social access to sufficient, safe and nutritious food that is suitable for all.

Problems of food insecurity are growing across the world, including in economically developed countries. In Europe, around half a million people do not have regular and sufficient access to food and about 20 million families cannot regularly afford high-quality meals (i.e., fish, meat or their vegetarian equivalents). These numbers are expected to rise due to the COVID-19 pandemic.

In this context, many countries recognize the importance of trade policies to ensure adequate levels of food security. Fusco et al. [6] analyzed the impact of trade opening on the level of food safety in European countries, using a dynamic panel analysis with the generalized moments method (GMM) approach.

Two different food safety indicators were selected (average protein intake and adequacy of average food energy supply) which provided information on both the quantity and nutritional quality of the food supply. In order to improve the robustness of the empirical results, three different regressions were developed, with three indicators of commercial openness (commercial opening, tariff and globalization) for each food safety indicator. The results showed that trade opening has, on average, a statistically significant positive impact on the food security of European countries.

Papers in this Special Issue, “Interdisciplinary Approach to Improve AgriFood Safety and Quality”, principally concern the prevention of human health risks by improving nutraceutical characteristics through the application of innovative analytical methods such as metabolomics and the use of genotoxic and mutagenic biomarkers.

We are happy to have discovered many new colleagues with similar research interests, using perhaps different definitions and methods of approaching the topic, but nevertheless sharing the devotion to food safety in the current globalized world, where it should be balanced with food security and risk assessment.
